# ﻿*Danxiaorchismangdangshanensis* (Orchidaceae, Epidendroideae), a new species from central Fujian Province based on morphological and genomic data

**DOI:** 10.3897/phytokeys.212.91534

**Published:** 2022-10-28

**Authors:** Miao Zhang, Xiao-Hui Zhang, Chang-Li Ge, Bing-Hua Chen

**Affiliations:** 1 College of Life Sciences, Fujian Normal University, Fuzhou 350117, China Fujian Normal University Fuzhou China; 2 The Public Service Platform for Industrialization Development Technology of Marine Biological Medicine and Products of the State Oceanic Administration, Fujian Key Laboratory of Special Marine Bioresource Sustainable Utilization, Southern Institute of Oceanography, College of Life Sciences, Fujian Normal University, Fuzhou 350117, China Fujian Normal University Fuzhou China

**Keywords:** Chloroplast genome, Epidendroideae, morphology, phylogeny, taxonomy

## Abstract

*Danxiaorchismangdangshanensis*, a new mycoheterotrophic species from Fujian Province, China, is described and illustrated. The new species is morphologically similar to *D.singchiana*, but its callus of labellum is a less distinctive Y-shape with three auricles on the apex, four pollinia that are narrowly elliptic in shape and equal in size, and it lacks fine roots. The plastome of *D.mangdangshanensis* is highly degraded. Phylogenetic analyses distinguished *D.mangdangshanensis* from its congeners, *D.singchiana* and *D.yangii*, with strong support based on nrITS + *matK* and plastomes, respectively.

## ﻿Introduction

The Orchidaceae, one of the largest families of angiosperms, were classified into five subfamilies based on their morphological and molecular characteristics, including Apostasioideae, Cypripedioideae, Vanilloideae, Orchidoideae, and Epidendroideae, with Epidendroideae being the largest (Chase et al. 2015). Identifying orchid species may be challenging, particularly during the vegetative stage when many orchid species plants exhibit very similar morphological characteristics. Moreover, many orchid species can crossbreed successfully across a wide range, giving rise to many intermediate types and natural variants. Therefore, phylogenetic analysis was more and more often employed to investigate the interrelationships among Orchidaceae species (Zhai et al. 2013; Lee et al. 2020; Li et al. 2020).

More than a few Epidendroideae species lack green leaves, resulting in reduced photosynthetic capacities and reliance on mycoheterotrophy for nourishment, i.e., indirectly exploiting other plants through mycorrhizal fungi (Brundrett 2009). Mycoheterotrophic plants, which are classified into two types, photosynthetic mycoheterotrophs and full mycoheterotrophs, are excellent examples of genomic modification due to relaxed selective constraints on photosynthetic function (Barrett et al. 2019). They possess distinct anatomical, physiological, and genomic features. One of these is a reduction in plastid genome size through loss or pseudogenization of photosynthesis-related genes. Early studies (Wolfe et al. 1992a; Wickett et al. 2008) suggested that the gene order of plastomes was conserved and that a large number of conserved genes were present; however, recent studies have revealed highly reduced (Delannoy et al. 2011; Wicke et al. 2013) and highly rearranged (Logacheva et al. 2014) plastomes. These findings indicate that mycoheterotrophic plants may have more diverse plastomes than previously thought.

*Danxiaorchis* (Calypsoinae, Epidendreae), a recently identified fully mycoheterotrophic orchid genus, was characterized by a distinct Y-shaped callus in its labellum. Only two species of *Danxiaorchis* have been documented, *D.singchiana* and *D.yangii* (Zhai et al. 2013; Yang et al. 2017). The plastid genome size of *D.singchiana* was found to have been dramatically reduced to 87, 910 dp (Li et al. 2020), however there is no plastome data available for its only congener, *D.yangii*.

In this paper, we describe a new orchid species found in Mangdang Mountain, Nanping City, in Fujian, China. The plant has a distinct morphology from the other known *Danxiaorchis* species. On the basis of morphological characteristics and molecular phylogenetic study, we propose a new species of *Danxiaorchis* and describe it below.

## ﻿Materials and methods

### ﻿Morphological description

The morphological description of the new species was based on the study of specimens collected in a variety of spots in 2022. A stereoscopic zoom microscope (Carl Zeiss, Axio zoom. v.16, Germany), equipped with an attached digital camera (Axiocam), and a digital caliper were used to record the sizes of the morphological characters. Field observations provided habitats and phenology for the new species.

### ﻿DNA extraction and sequencing

In this study, total DNA was extracted from freeze-dried gynostemium and the ovary of the new species using a DNeasy Plant Mini Kit (Qiagen, Valencia, CA, USA). The phylogenetic position of the new species was determined by *nr*ITS and plastid *matK* sequences. The nrITS (18S-ITS1-5.8S-ITS2-26S) was assembled using GetOrganelle v1.7.5, with -R of 7 and k-merset of “35, 85, 115”, the embplant_nr library was selected as the reference genome database, then annotated and visualized using Geneious v2021.2.2. The plastid *matK* was extracted from the genome sequence via Geneious v.2021.2.2.

### ﻿Genome sequencing, assembly, annotation and analysis

Purified total DNA of the new species was fragmented, genome skimming was performed using next-generation sequencing technologies on the Illumina Novaseq 6000 platform with 150 bp paired-end reads and 480 bp insert size by Berry Genomics Co. Ltd. (Beijing, China), and 15.88 GB of reads was obtained.

The paired-end reads were filtered and assembled into complete plastome using a GetOrganelle v1.7.5.0 (Jin et al. 2020a) with appropriate parameters, with K-merset “21,45,65,85,105”, the word size is 0.6. Following previous studies, our workflow includes five key steps as well: 1. Mapping reads to seed and assembling seed-mapped reads for parameter estimation; 2. Recruiting more target-associated reads through extending iterations; 3. Conducting de novo assembly; 4. Roughly filtering fortarget-like contigs; 5. Identifying target contigs and exporting all configurations (Camacho et al. 2009; Bankevich et al. 2012; Langmead and Salzberg 2012; Jin et al. 2020). Graphs of the final assembly were visualized by Bandage (Wick et al. 2015) to assess their completeness. Gene annotation was performed using CPGAVAS2 (Shi et al. 2019) and PGA (Qu et al. 2019). The different annotations of protein coding sequences were confirmed using BLASTx. The tRNAs were checked with tRNAscan-SE v2.0.3. Final chloroplast genome maps were created using OGDRAW.

### ﻿Phylogenetic analysis

The phylogenetic relationship was constructed using Maximum likelihood (ML) and Bayesian Inference (BI) analyses with the combined ITS and *matK* sequences. In total, 39 samples of *Calypso*, *Changnienia*, *Chysis*, *Corallorhiza*, *Cremastra*, *Dactylostalix*, *Danxiaorchis*, *Ephippianthus*, *Govenia*, *Tipularia*, *Yoania* and *Yunorchis* were included in our analysis. A species of *Chysis* was used as outgroup. Each individual locus was aligned using MAFFT 7.310 (Katoh and Standley 2013) with default settings. A concatenated supermatrix of the ITS sequences and matK was generated using PhyloSuitev.1.1.15 (Zhang et al. 2019) for the phylogenetic analysis. All missing data were treated as gaps. The best nucleotide substitution model according to the Bayesian Information Criterion (BIC) was K3Pu+F+R2, which was selected by ModelFinder (Kalyaanamoorthy et al. 2017) implemented in IQTREE v.1.6.8. Maximum likelihood phylogenies were inferred using IQ-TREE (Nguyen et al. 2015) under the model automatically selected by IQ-TREE (‘Auto’ option in IQ-TREE) for 2000 ultrafast (Minh et al. 2013) bootstraps. Bayesian Inference phylogenies were inferred using MrBayes 3.2.6 (Ronquist et al. 2012) under the GTR+F+G4 model (2 parallel runs, 2000000 generations). Phylograms were visualized in iTOLv.5 (iTOL: Interactive Tree Of Life (embl.de)).

To construct a phylogenetic tree based on plastome sequences, a total of 20 plastome sequences of *Calypso*, *Corallorhiza*, *Cremastra*, *Danxiaorchis*, *Cattleya*, *Anathallis*, *Masdevallia*, *Neofinetia* and *Calanthe* were included. Among them, *Calypso*, *Corallorhiza*, *Cremastra* and *Danxiaorchis* belong to Calypsoinae; *Cattleya* belongs to *Laeliinae*; and *Anathallis* and *Masdevallia* belong to *Pleurothallidinae*. *Neofinetiafalcata* and *Calanthetriplicata* were used as outgroups. Each individual locus was aligned using MAFFT 7.310 (Katoh and Standley 2013) with default settings. The best nucleotide substitution model according to the Bayesian Information Criterion (BIC) was TVM+F+R4, which was selected by ModelFinder (Kalyaanamoorthy et al. 2017) implemented in IQTREE v.1.6.8. Maximum likelihood phylogenies were inferred using IQ-TREE (Nguyen et al. 2015) under the model automatically selected by IQ-TREE (‘Auto’ option in IQ-TREE) for 2000 ultrafast (Minh et al. 2013) bootstraps. Bayesian Inference phylogenies were inferred using MrBayes 3.2.6 (Ronquist et al. 2012) under the GTR+F+I+G4 model (2 parallel runs, 2000000 generations), in which the initial 25% of sampled data were discarded as burn-in. Phylograms were visualized in iTOLv.5 (iTOL: Interactive Tree Of Life (embl.de)).

## ﻿Results

### ﻿Comparative analysis of the plastomes

The plastome of *Danxiaorchismangdangshanensis* was compared to those of the other 18 species in the subtribe Epidendreae. The plastome size of these species varied greatly from 85,273 bp in *D.mangdangshanensis* to 157,423 bp in *Masdevalliacoccinea* (a photosynthetic orchid) (Table 1), with the new species being the smallest. The length of the IR region of *D.mangdangshanensis* was the shortest across all compared species, while the length of the LSC region was slightly longer than that of *D.singchiana*, but shorter than the remaining species studied. The SSC region of *D.mangdangshanensis* was intermediate in length compared to those of the other orchid species. The plastome size of mycoheterotrophic species showed high correlation with the size of both the SSC and IR.

**Table 1.** Statistics on the basic features of the plastid genomes of *Danxiaorchismangdangshanensis* and related taxa.

Species	Accession No.	Voucher	Number of Genes			Length (bp)				GC Content (%)			
			PCGs	tRNA	rRNA	Total	LSC	SSC	IR	Total	LSC	SSC	IR
*Danxiaorchismangdangshanensis*	OP122564	Huang &Chen	32	20	4	85,273	42,605	18,766	11,951	34.41	30.84	37.95	37.99
*Danxiaorchissingchiana*	MN990438	Jin	29	22	4	87,910	42,494	17,890	13,763	34.55	31.12	39.01	36.97
Calypsobulbosavar.occidentalis	MG874037	CFB	71	30	4	149,313	84,543	14,846	24,962	37.13	34.54	29.36	43.52
*Corallorhizabentleyi*	MG874035	Freudenstein 2550	52	31	4	124,482	64,420	10,722	24,670	36.60	32.62	25.81	42.94
*Corallorhizabulbosa*	KM390013	–	68	30	4	148,643	83,422	15,343	24,939	37.14	34.31	29.16	43.37
*Corallorhizamacrantha*	KM390017	Salazar A	66	30	4	151,031	84,262	12,545	27,112	37.21	34.42	29.38	43.35
*Corallorhizamertensiana*	KM390018	Freudenstein 1999	54	30	4	147,941	81,109	13,774	26,529	36.78	33.92	28.10	43.41
*Corallorhizaodontorhiza*	KM390021	–	67	30	4	147,317	82,259	13,508	25,775	36.99	34.24	28.28	43.66
*Corallorhizastriata*	MG874034	CFB	47	29	4	141,202	75,701	13,319	26,091	36.34	33.12	27.33	43.27
*Corallorhizatrifida*	MG874036	Freudenstein 2763a	67	30	4	149,376	83,685	15,285	25,203	37.21	34.55	28.99	43.75
*Corallorhizawisteriana*	KM390020	Freudenstein 2462	67	30	4	146,437	82,350	11,743	26,172	37.05	34.27	28.11	43.43
*Cremastraappendiculata*	MG925366	–	73	30	4	155,320	87,098	15,478	26,372	37.19	34.55	30.41	43.54
*Cattleyacrispate*	KP168671	–	71	30	4	148,343	86,254	13,261	24,614	37.26	34.88	29.35	43.36
*Cattleyaliliputana*	KP202881	–	71	30	4	147,092	85,804	13,900	23,694	37.35	34.88	30.19	43.45
*Anathallisobovata*	MH979332	UPCB:M.C. Santos	81	30	4	155,515	83,694	20,047	25,542	37.05	34.65	30.05	43.10
*Masdevalliacoccinea*	KP205432	–	79	30	4	157,423	84,957	18,448	27,009	36.81	34.42	29.44	43.10
*Masdevalliapicturata*	KJ566305	–	80	29	4	156,045	85,145	20,742	25,079	36.88	34.44	29.74	43.22
*Neofinetiafalcate*	KT726909	PDBK	67	30	4	156,045	84,948	18,029	26,534	36.64	34.44	29.74	43.22
*Calanthetriplicata*	KF753635	–	80	30	4	132,271	87,263	18,476	26,510	36.74	34.40	29.73	43.03

### ﻿Phylogenetic analysis

**Figure 1.**
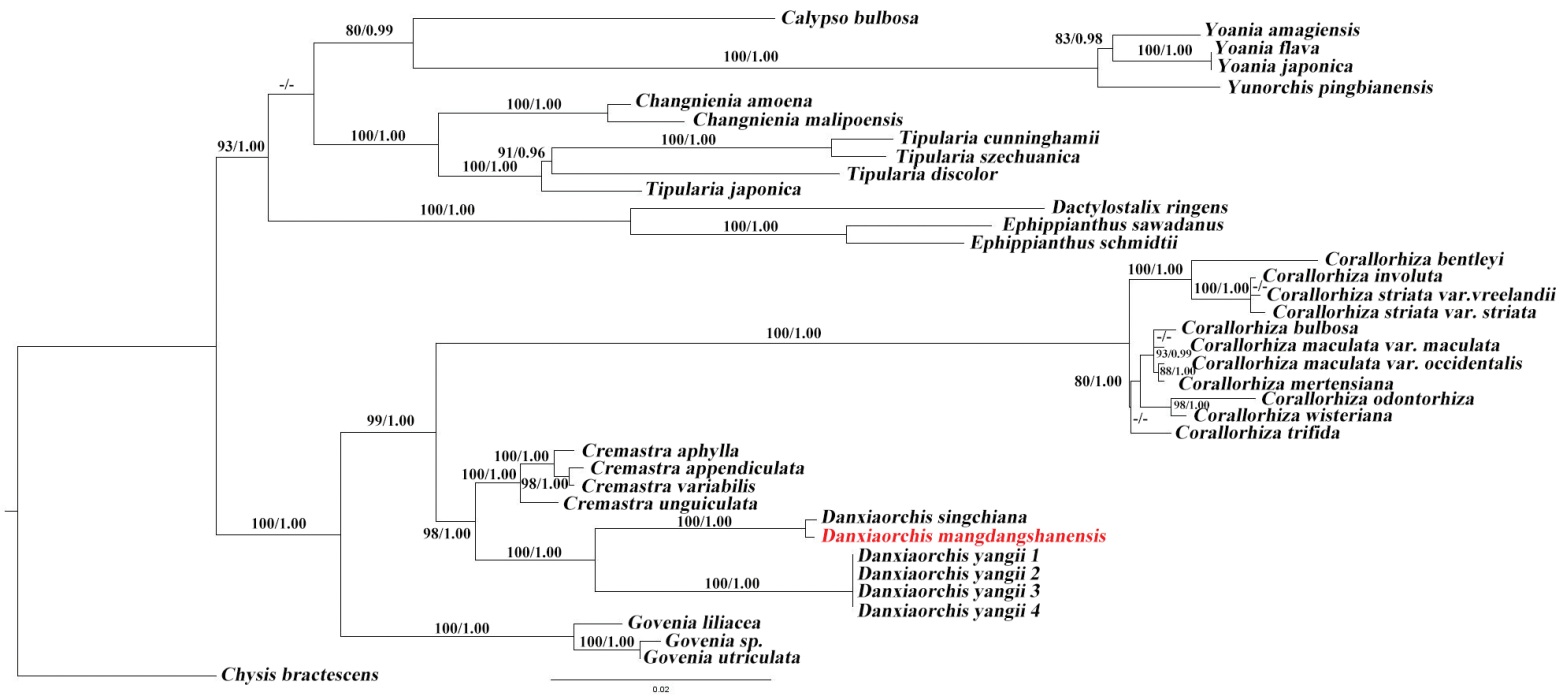
Phylogenetic tree of orchid species in the subtribe Epidendreae based on Bayesian Inference of nrITS and *matK* sequences. Numbers above and below branches indicate RAxML (left) bootstrap probabilities (BP) and Bayesian (right) posterior probabilities (PP), respectively.

**Figure 2.**
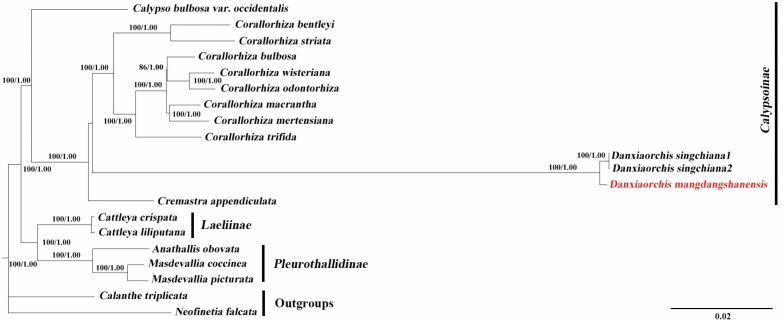
Phylogenetic tree of orchid species in the subtribe Epidendreae based on Bayesian Inference of whole plastomes. Numbers above and below branches indicate RAxML (left) bootstrap probabilities (BP) and Bayesian (right) posterior probabilities (PP), respectively.

Phylogenetic relationships were first reconstructed by Maximum likelihood (ML) and Bayesian Inference (BI) analyses using combined ITS and *matK* sequences, as well as the plastome data. The nrITS and *matK* tree (Fig. 1) clearly indicated the distinctiveness of *Danxiaorchismangdangshanensis* from its two congeners, *D.singchiana* and *D.yangii*, with strong support (PP = 1, BS = 100), and the new species is closer to *D.singchiana*. *Danxiaorchis* is sister to *Cremastra*, which is consistent with previous studies (Freudenstein et al 2017; Li et al. 2019; 2020). In addition, the phylogenetic analysis based on entire plastomes also separates the new species from *D.singchiana* with strong support (PP = 1, BS = 100) (Fig. 2).

## ﻿Taxonomic treatment

### ﻿ Danxiaorchis mangdangshanensis

Taxon classificationPlantaeAsparagalesOrchidaceae

Q. S. Huang, Miao Zhang, B. Hua Chen & Wang Wu sp. nov.

4BD3A41B-9DBE-5F8C-887D-6A43FD209FAC

urn:lsid:ipni.org:names:77307479-1

Figs 3 , 4 , 5

#### Diagnosis.

*Danxiaorchismangdangshanensis* can be easily distinguished from *D.singchiana* by having no fine roots, fewer flowers in the raceme, the side lobes of the labellum are ivory-white rather than yellow, and it has only 3 colored strips rather than 4–5 pairs. Additionally, its callus is a less distinctive Y-shape and has three auricles, with a purple-red spot on each auricle at the front, and the callus has a remarkable striped appendage adaxially. Furthermore, there are narrow wings on the side of column, and the four pollinia are narrowly elliptic in shape and equal in size.

**Figure 3.**
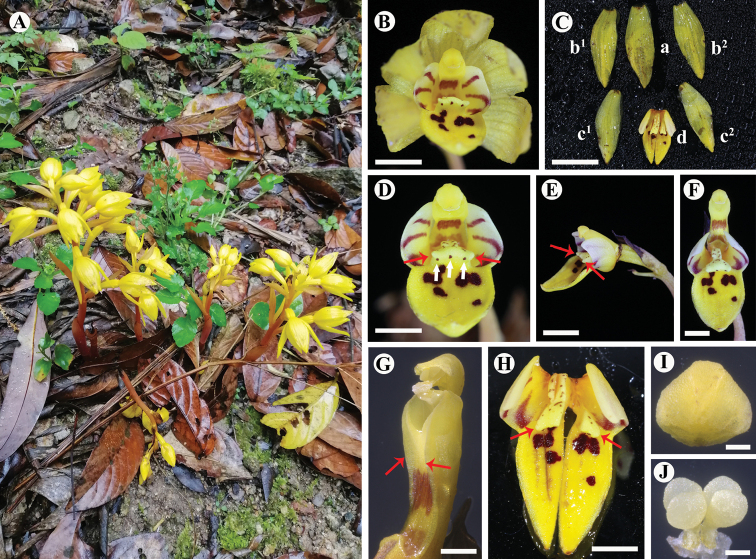
*Danxiaorchismangdangshanensis* Q. S. Huang, Miao Zhang, B. Hua Chen & Wang Wu, sp. nov. **A** flowering and habitat (photographed by Wang Wu) **B** front view of a flower **C-a** dorsal sepal **C-b** lateral sepals **C-c** petals **C-d** labellum **D** gynostemium and labellum, front view, showing three purple-red spots (white arrows) on the Y-shaped callus (red arrows) **E** gynostemium and labellum, side view, showing three auricles(red arrows) **F** labellum, showing remarkable striped appendage **G** gynostemium, showing narrow wings on the both sides (red arrows) **H** cross section of labellum, showing indistinct Y-shaped callus (red arrows) **I** anther cap **J** pollinarium, front view, showing pollinia 4 in 2 pairs. Scale bars: 5 mm (**B**); 1 cm (**C**); 4 mm (**D**); 5 mm (**E**); 4 mm (**F**); 1 mm (**G**); 4 mm (**H**); 500 μm (**I, J**).

#### Type.

China. Fujian (福建) Province, Nanping (南平) City, Yanping (延平) District, Mangdangshan Mountain, Mangdangshan National Nature Reserve, forest margins, 26°41'N, 118°2'E, elevation 375 m, 5 May 2022, Q.S. Huang & B. Hua Chen *CBH 04593* (Holotype, FNU, barcode FNU0041324; Isotypes, FNU, barcode FNU0041325).

**Figure 4.**
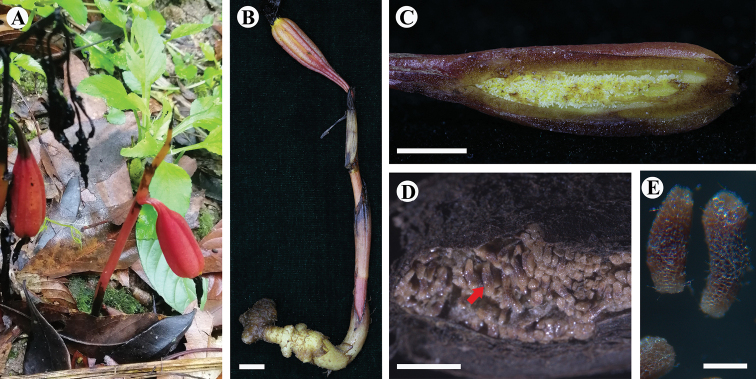
*Danxiaorchismangdangshanensis* Q. S. Huang, Miao Zhang, B. Hua Chen & Wang Wu, sp. nov. **A** fruit-bearing plant (photographed by Wang Wu) **B** infructescence and rhizome **C** longitudinal section of immature capsule **D** mature capsule, showing mature seeds (red arrow) **E** mature seeds. Scale bars: 1 cm (**B, C**); 1 mm (**D, E**).

#### Description.

Plant erect, 10.6–22.2 cm tall, holomycotrophic. Rhizome tuberous, fleshy, cylindrical, 2.5–5.3 cm long, 7.0–11.2 mm thick, with short branches, 4.5–5.6 mm long, without roots. Scape terete, pale red-brown, 4.2–5.8 mm thick, 3-sheathed; sheaths cylindrical, clasping stem, membranous, 16.2–43.4 × 4.5–8.7 mm. Inflorescence racemose, 2.9–9.6 cm long, 4- to 10-flowered; floral bracts oblong-lanceolate, 10.5–29.8 × 3.0–11.1 mm, apex acuminate, pale yellow; pedicel and ovary bright yellow,13.8–22.9 mm long, glabrous; sepals yellow, obovate elliptic, dorsal sepals 13.5–17.2 × 4.8–6.5 mm, obtuse; lateral sepals 16.3–18.6 × 5.9–6.7 mm, obtuse; petals yellow, narrowly elliptic, 15.5–19.7 × 6.0–6.5 mm, acute; labellum 3-lobed, with 3 pairs of purple-red stripes on side lobes and purple-red spots on middle lobe; side lobes erect, ivory-white, slightly clasping the column, subsquare, 4.5–5.6 × 5.3–6.2 mm; mid-lobe oblong, 7.8–10.2 × 6.1–7.8 mm, apex acute to obtuse; labellum with two sacs at the base and a fleshy callus centrally, indistinctive Y-shaped (in the transition to “T-shape”), with 3 auricles on the apex, each of which has 1 purple-red spot at the front; callus extending from the base of disc to the base of mid-lobe, triangular at the base of mid-lobe, fleshy, ca. 3.1 mm wide, 0.25 m long, narrows into a raised band when extended, ca. 1.5 mm wide, 0.4 mm long, with sparse purple-red spots; column cream colored, straight, semi-cylindrical, narrow wings on the side, 4.9–6.3 mm long, 2.9 mm wide, footless; stigma concave, triangular, terminal; anther cap ellipsoid, ca. 1.3 mm in diameter; pollinia four, in two pairs, narrowly elliptic, granular-farinaceous, composed of friable massulae, each pair containing two pollinia equal in size with a thick caudicle attached to a common subsquare viscidium, ca. 0.5 mm in diameter. Capsule purple red, fusiform, 3 evident banded ridges, 37.3–46.8 mm long, 8.9–10.1 mm thick. Seeds light dark brown, cylindrical, 1.3 × 0.3 mm, fleshy, honeycombed stripes on the seed coat surface.

**Figure 5.**
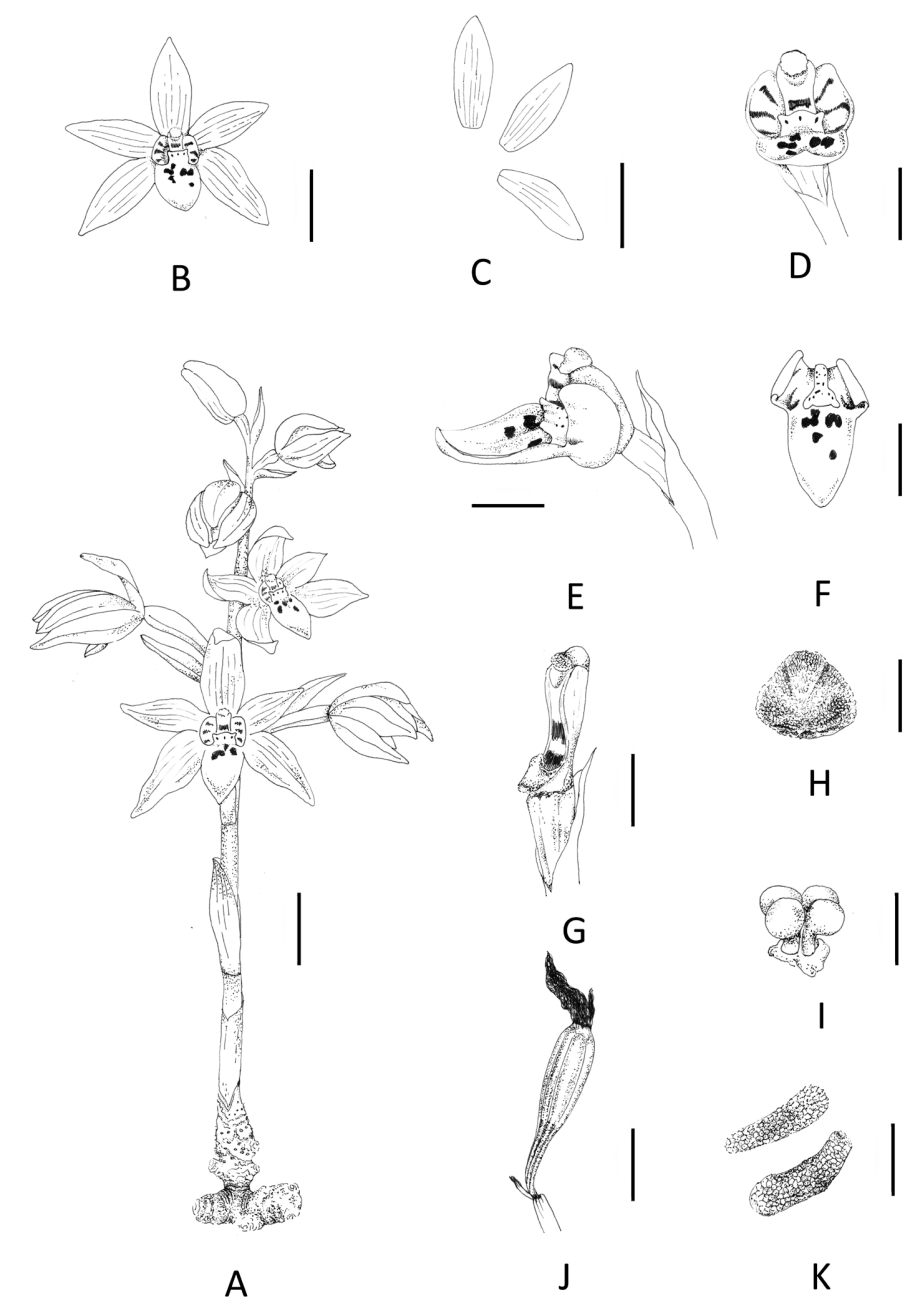
*Danxiaorchismangdangshanensis* Q. S. Huang, Miao Zhang, B. Hua Chen & Wang Wu, sp. nov. **A** flowering plant **B** flower, front view **C** dissection of a flower, showing dorsal sepal, petal, lateral sepal **D** gynostemium and labellum, front view **E** gynostemium and labellum, side view **F** labellum **G** gynostemium **H** anther cap **I** pollinarium **J** immature capsule **K** mature seeds. Scale bars: 1.0 cm (**A, B, C, J**); 0.5 cm (**D–F**); 0.2 cm (**G**); 1.0 mm (**H, I, K**).

#### Distribution and habitat.

**Figure 6.**
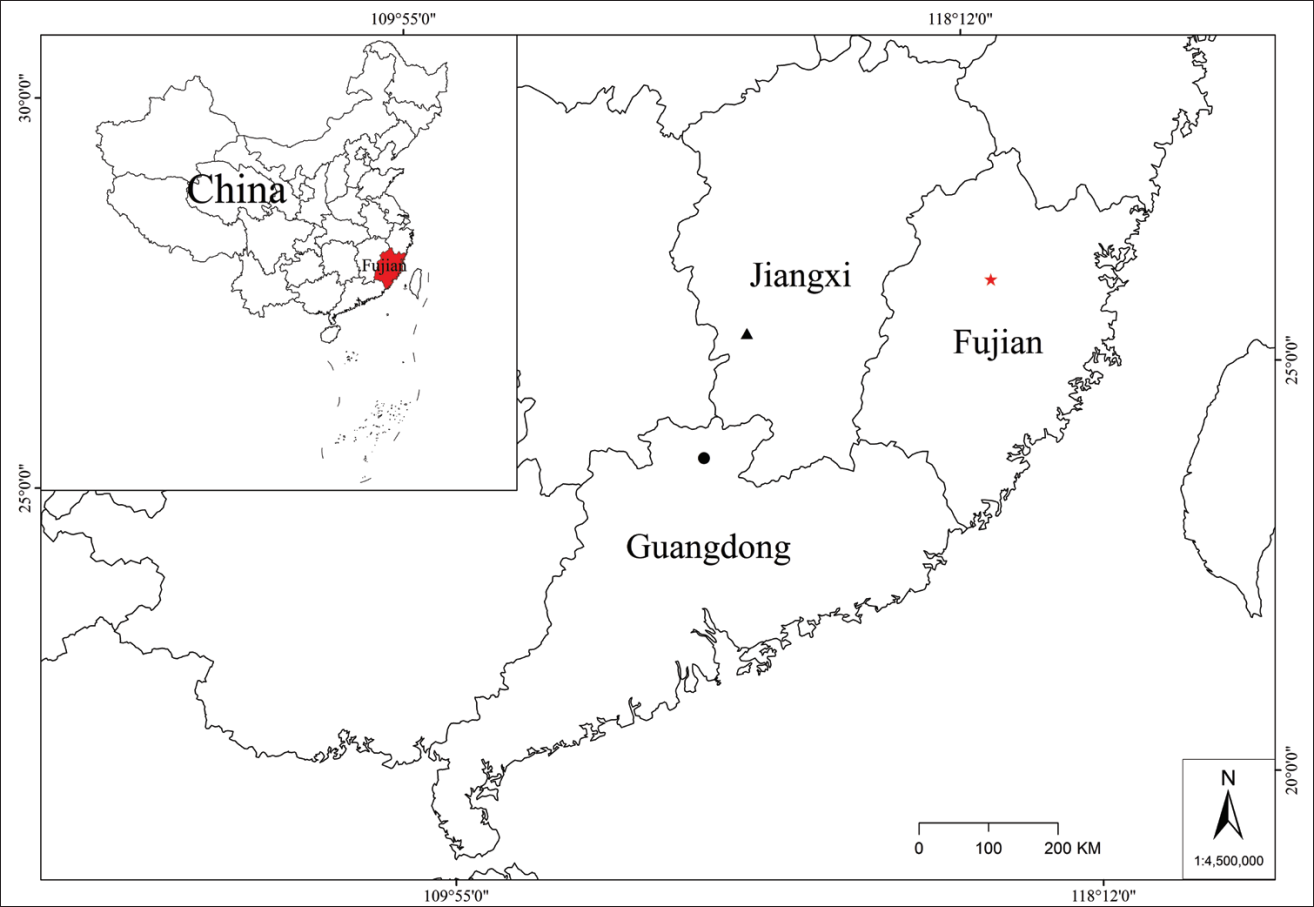
Distribution of *Danxiaorchismangdangshanensis*, *D.singchiana*, *D.yangii* in China. Legend: (red star) *D.mangdangshanensis*, (black circle) *D.singchiana*, (black triangle) *D.yangii*.

*Danxiaorchismangdangshanensis* is only found in Mangdangshan National Nature Reserve, Fujian, China (Fig. 6), where it grows at the margin of mid-subtropical evergreen broad-leaved forest, beside a canal near a *Musabalbisiana* forest. Many other plants grow in the surrounding habitat, whose tree layer includes *Castanopsisfargesii* Franch. (Fagaceae), *C.fissa* (Champion ex Bentham) Rehder et E. H. Wilson (Fagaceae), and *Verniciamontana* Lour. (Euphorbiaceae); the shrub layer includes *Ficuserecta* Thunb. (Moraceae), *F.hirta* Vahl (Moraceae), *Maesajaponica* (Thunb.) Moritzi. ex Zoll. (Primulaceae), *Callicarpakochiana* Makino (Lamiaceae), and *Aucubachinensis* Benth. (Garryaceae); the vegetation layer includes *Angiopterisfokiensis* Hieron. (Marattiaceae), *Violadiffusa* Ging. (Violaceae), *Mazusfukienensis* Tsoong (Mazaceae), *Gynostemmapentaphyllum* (Thunb.) Makino (Cucurbitaceae), *Irisjaponica* Thunb. (Iridaceae), *Musabalbisiana* Colla (Musaceae), and *Miscanthusfloridulus* (Lab.) Warb. ex Schum et Laut. (Poaceae); the interlayer plants include *Fissistigmaoldhamii* (Hemsl.) Merr. (Annonaceae), and StauntoniaobovatifoliolaHayatasubsp.urophylla (Hand.-Mazz.) H.N.Qin (Lardizabalaceae).

#### Phenology.

Flowering was observed from mid-April to early May, and fruiting from mid-May to mid-June.

#### Etymology.

The *Mang dang shan dang xia lang* (茫荡山丹霞兰).The epithet *mangdangshanensis* (茫荡山) refers to Mangdangshan Mountain, Mangdangshan National Nature Reserve, Fujian Province where this new species was found.

#### Conservation status.

During our fieldwork in 2022, three populations of about 14 plants of the new species were found in Mangdangshan National Nature Reserve, Fujian Province, China. And hence, we suggest its placement in the Data Deficient category of IUCN (2022). According to the Updated List of National Key Protected Wild Plants (Decree No. 15) by the country’s State Forestry and Grassland Administration and the Ministry of Agriculture and Rural Affairs, *Danxiaorchis* are classified in the national secondary protection list. The new recorded genus should also be included on the national secondary protection list during the upcoming revision process.

##### ﻿Characteristics of the *Danxiaorchismangdangshanensis* plastome

**Figure 7.**
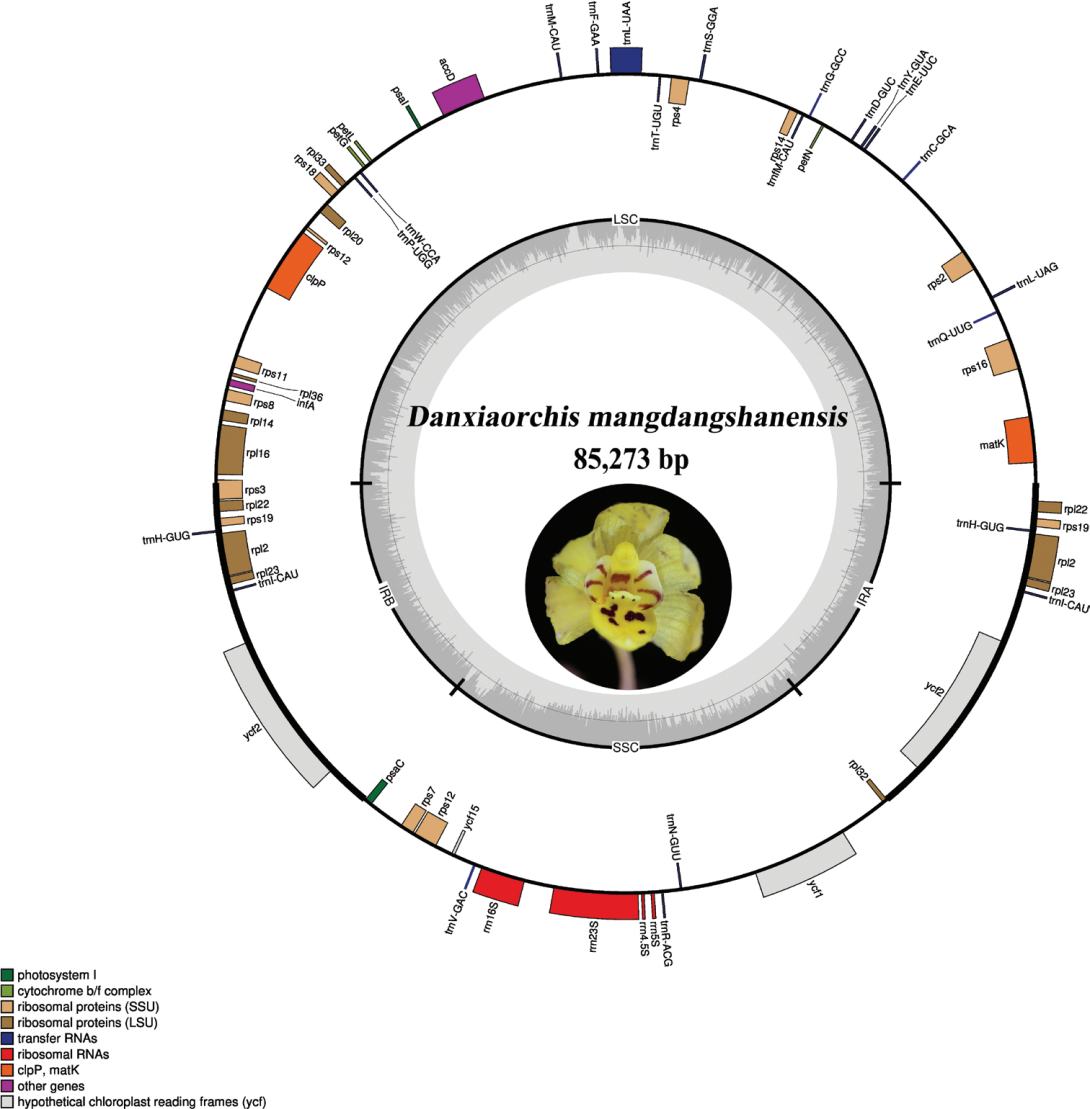
Circular gene map of the plastid genome of *Danxiaorchismangdangshanensis*. Genes inside the circle are transcribed clockwise, while those drawn outside are transcribed counterclockwise. Genes are color-coded according to their functional groups. The circle inside the GC content graph marks the 50% threshold.

The highly reduced plastid genome of *Danxiaorchismangdangshanensis* still has a quadripartite structure and is 85,273 bp with a large single-copy (LSC) region of 42,605 bp separated from a small single-copy (SSC) region of 18,766 bp by two inverted repeat regions (IRs), each of 11,951 bp (Fig. 7). A total of 56 unique genes were identified in the plastome and it contains 32 protein-coding genes, 20 tRNAs, and four rRNAs. A total of seven genes were duplicated in the IR regions, including *rpl22*, *rps19*, *trnH-GUG*, *rpl2*, *rpl23*, *trnI-CAU*, *ycf2* (Table 2). The total GC content of the plastome is 34.40%. Two inversions were detected in the plastome of *D.mangdangshanensis* (Suppl. material 1: Fig. S1), which are also reported for *D.singchiana* (Li et al. 2020). The annotated plastome was deposited in GenBank (accession number OP122564).

**Table 2.** Gene contents in the plastid genome of *Danxiaorchismangdangshanensis*.

Category, group of Genes	Gene names
**Photosynthesis**:	
Subunits of photosystem I	*psaC*, *psaI*
Subunits of photosystem II	–
Subunits of NADH dehydrogenase	–
Subunits of cytochrome b/f complex	*petG*, *petL*, *petN*
Subunits of ATP synthase	–
Large subunit of rubisco	–
Subunits photochlorophyllide reductase	–
**Self-replication**:	
Proteins of large ribosomal subunit	*rpl14*, rpl16*, rpl2*(2), *rpl20*, *rpl22(2)*, *rpl23(2)*, *rpl32*, *rpl33*, *rpl36*
Proteins of small ribosomal subunit	*rps11*, rps12**, *rps14*, rps16*, *rps18*, *rps19* (2), *rps2*, *rps3*, *rps4*, *rps7*, *rps8*
Subunits of RNA polymerase	–
Ribosomal RNAs	*rrn16S*, *rrn23S*, *rrn4.5S*, *rrn5S*
Transfer RNAs	*trnC-GCA*, *trnD-GUC*, *trnE-UUC*, *trnF-GAA*, *trnG-GCC*, *trnH-GUG (2)*, *trnI-CAU (2)*, trnL-UAA*, *trnL-UAG*, *trnM-CAU*, *trnN-GUU*, *trnP-UGG*, *trnQ-UUG*, *trnR-ACG*, *trnS-GGA*, *trnT-UGU*, *trnV-GAC*, *trnW-CCA*, *trnY-GUA*, *trnfM-CAU*
**Other genes**:	
Maturase	*matK*
Protease	clpP**
Envelope membrane protein	–
Acetyl-CoA carboxylase	*accD*
c-type cytochrome synthesis gene	–
Translation initiation factor	i*nfA*
**Genes of unknown function**:	
Conserved hypothetical chloroplast ORF	*ycf1*, *ycf15*, *ycf2(2)*

Notes: *gene with one introns; **gene with two introns; #Pseudo gene; Gene (2): Number of copies of multi-copy genes.

## ﻿Discussion

The characteristic Y-shaped callus on its labellum clearly indicates the new species *Danxiaorchismangdangshanensis* belongs to the genus *Danxiaorchis*, and this conclusion was strongly supported by phylogenetic analyses based on combined datasets of ITS and *matK*, as well as the whole plastome. On the basis of a comprehensive morphological comparison, the new species can be distinguished from its two congeners, *D.singchiana* and *D.yangii* (Table 3). It was noticeable that the callus of *D.mangdangshanensis* had a less distinctive Y-shape, with three auricles on the apex, and a purple-red spot on each auricle at the front. Adaxially, the callus features a unique striped appendage. The Y-shaped callus of *D.yangii* was remarkably large and had an obovoid appendage at its base adaxially (Yang et al. 2017). Among the mycoheterotrophic taxa of Epidendroideae, *Danxiaorchis* have four sectile pollinia that are granular-farinaceous, with distinct caudicles and viscidium (Zhai et al. 2013; Yang et al. 2017). This configuration is unique in Epidendreae (i.e., *Yoania*; Chen et al. 2009), and its possible taxonomic significance awaits further study.

**Table 3.** Morphological and distribution altitude differences between *Danxiaorchismangdangshanensis*, *D.singchiana* and *D.yangii*.

Characteristics	*D.mangdangshanensis*	*D.singchiana*	*D.yangii*
Roots	Branches, no fine roots	Fine roots and branches	Fine branches, no fine roots
Flowers in the raceme	4–10	6–18	5–30
Color of side lobes of labellum	Ivory-white	Yellow	Yellow
Number of stripes on the side lobes	3	4–5	3
callus	Indistinctive Y-shaped, three auricles at the front	Distinctive Y-shaped	Y-shaped, remarkable large
Front view of the callus	3 distinct purple-red spots	None	None
Callus adaxially bearing	A remarkable striped appendage	An obovoid appendage	A remarkable obovoid appendage
Size of four pollinia	Equal in size	Different in size	Equal in size
Narrow wings on the side of the stamen column	Yes	No	No
Distribution altitude/m	ca. 370	ca.130	ca. 360

The plastome of *Danxiaorchismangdangshanensis* was compared to those of the other 18 species in the subtribe Epidendreae. Although the genome sizes of the investigated species varied greatly, they all possessed typical quadripartite structures. This variance in genome size was mostly caused by variations in the length of the IR and SSC regions. The plastome of *Danxiaorchis* is more “degraded” than those of the other orchid species in the tribe Epidendreae examined, which is mostly due to gene losses associated with mycoheterotrophic habitats. However, the 15 essential genes among orchid plastomes to maintain minimal plastome activity (Kim et al. 2020) were all present in the plastome of *D.mangdangshanensis*, including the three subunits of *rpl* (14, 16, and 36), seven subunits of *rps* (2, 3, 4, 7, 8, 11, and 14), three subunits of *rrn* (5s, 16s, and 23s), *trn*C-GCA, and *clp*P genes. The IR region of *D.mangdangshanensis* was half that of most orchid species studied, and even smaller than its congener, *D.singchiana* (Li et al. 2020). The IR region plays a role in the structural stability of plastomes and its expansion or contraction due to changes in the amount of repeated DNA and/or changes in sequence complexity (Palmer and Thompson 1982).

The loss of the plastid genes within heterotrophic lineages occurred in a general order. The first was the loss of the NADH dehydrogenase-like (*ndh*) complex, which may frequently trigger irreversible evolutionary cascade losses of photosynthetic genes (*atp, psa*/*psb*, *pet*, *rbcL*, *ycf3*, *4*) and a plastid-encoded RNA polymerase (*rpo*). Followed by the loss of housekeeping genes involved in basic organellar functions such as intron splicing and translation (*rpl*, *rps*, *rrn*, *trn*, *accD*, *clpP*, *matK*, *ycf1*, 2) (Barrett and Davis 2012; Kim et al. 2020). In *Danxiaorchismangdangshanensis*, the *ndh* genes have completely disappeared, which is common in mycoheterotrophic orchids. This is interesting because they are also lost or become pseudogenized in photosynthetic orchids, such as *Oncidium* (Wu et al. 2010) and *Phalaenopsis* (Chang et al. 2006), raising questions about their significance to photosynthetic chloroplasts. In addition, nearly all photosynthetic genes and the *rpo* gene were lost in *D.mangdangshanensis*, representing whole-organismal loss of photosynthetic functions, which thus is a major transitory event in both physiology and genome evolution of the plant. *Ycf3* and *ycf4*, which are crucial to photosystem polypeptide function, were lost in *D.mangdangshanensis*, although the former was present but has become pseudogene in *D.singchiana* (Li et al. 2020). Furthermore, several housekeeping genes, including *rps15* and some *trn* genes, were lost in *D.mangdangshanensis*, which might be due to the increasing dependence on external carbon. It has been hypothesized that perhaps only a few loci, such as tRNA-Glu, tRNA-fMet, are absolutely essential in heterotrophic plants (Barbrook et al. 2006).

Plastid genome evolution in mycoheterotrophic lineages should be of concern in relation to the conservation of these plants, as many of them are rare or endangered (Leake 1994; Merckx and Freudenstein 2010). The mycoheterotrophs represent replicated evolutionary experiments in the loss of photosynthetic function, and its effect on genome evolution. It is evident that photosynthesis-related genes are the first to become pseudogenes or to be deleted in heterotrophic plants (Wolfe et al. 1992b; Wickett et al. 2008; Delannoy et al. 2011; Barrett and Davis 2012; Logacheva et al. 2014; Li et al. 2020). In spite of this, a number of questions remain unanswered regarding the evolution of heterotrophic plastomes, and the current study provides new information on these issues.

## Supplementary Material

XML Treatment for Danxiaorchis mangdangshanensis

## Supplementary materials

Supplementary material 1 Appendix S1 Data type Docx file. Explanation note **Figure S1.** The two inversions in the plastome of *Danxiaorchismangdangshanensis*. **Table S1.** GenBank information for the taxa used in the present study (matK and nrITS). **Table S2.** GenBank information for the taxa used in the present study (plastid genome). https://binary.pensoft.net/file/762759This dataset is made available under the Open Database License (http://opendatacommons.org/licenses/odbl/1.0/). The Open Database License (ODbL) is a license agreement intended to allow users to freely share, modify, and use this Dataset while maintaining this same freedom for others, provided that the original source and author(s) are credited.Miao Zhang, Xiao-Hui Zhang, Chang-Li Ge, Bing-Hua Chen
