# Supplementary material for: ﻿Danxiaorchismangdangshanensis (Orchidaceae, Epidendroideae), a new species from central Fujian Province based on morphological and genomic data
Source: PhytoKeys. 2022 Oct 28;212:37–55. doi: 10.3897/phytokeys.212.91534 (PMC9836500; doi:10.3897/phytokeys.212.91534)
Supplement: Supplementary material 1 — Appendix S1 [file phytokeys-212-037_article-91534__-s001.docx]

**Supplementary material 1**

**Appendix**

**
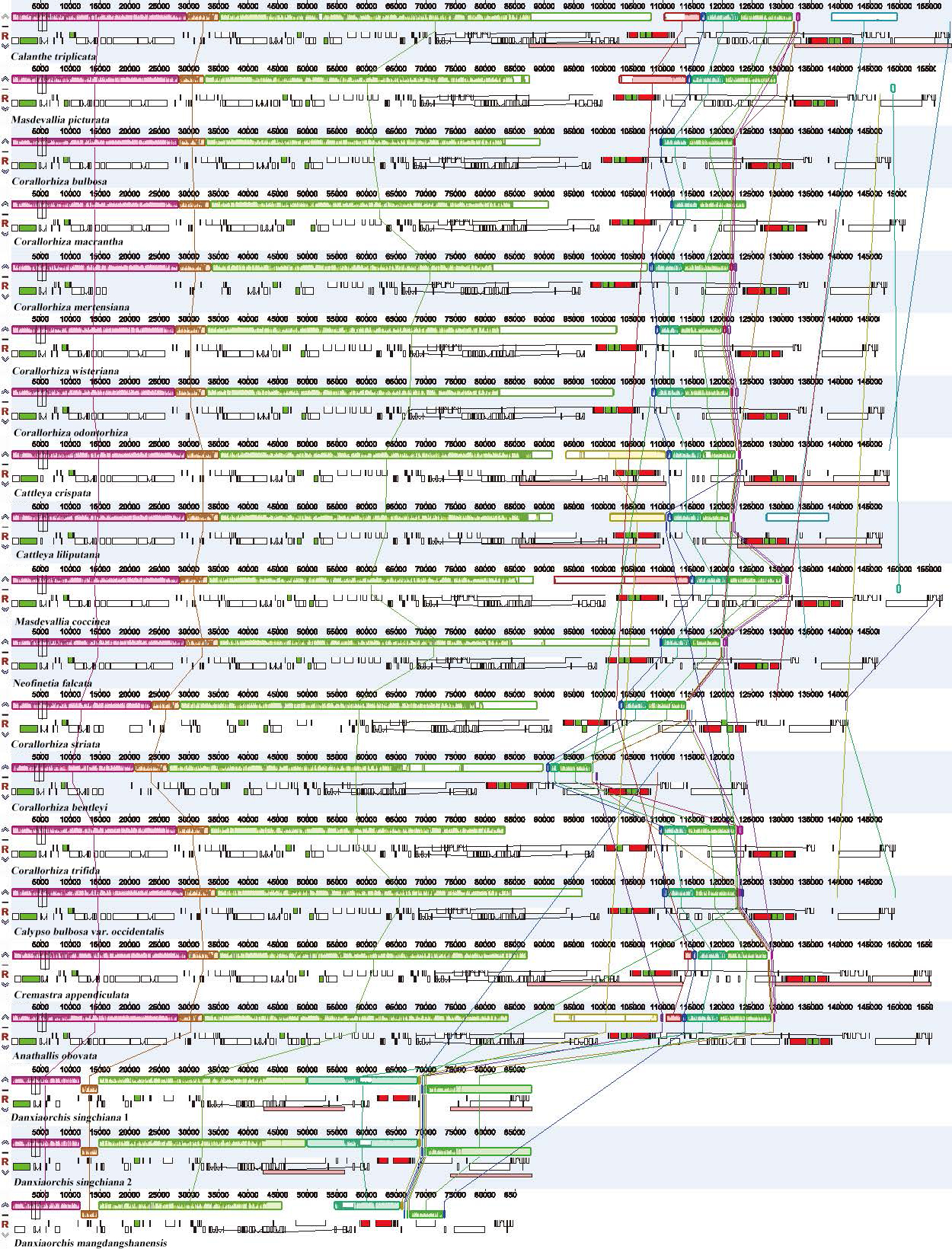
**

**Figure S1. The two inversions in the plastome of *Danxiaorchis mangdangshanensis*.**

Table S1. GenBank information for the taxa used in the present study (*matK* and nrITS)

| Number | Taxa | nrITS | *matK* |
| --- | --- | --- | --- |
| 1 | *Calypso bulbosa* | LC176591 | LC176612 |
| 2 | *Changnienia amoena* | LC176592 | LC176613 |
| 3 | *Changnienia malipoensis* | JX293179 | JX293183 |
| 4 | *Chysis bractescens* | EF079363 | AY121747 |
| 5 | *Corallorhiza bentleyi* | JF319668 | EF525706 |
| 6 | *Corallorhiza bulbosa* | EU391332 | EF525699 |
| 7 | *Corallorhiza involuta* | EU391347 | EF525698 |
| 8 | *Corallorhiza maculata var. maculata* | EU391329 | EF525700 |
| 9 | *Corallorhiza maculata var. occidentalis* | EU391330 | EF525697 |
| 10 | *Corallorhiza mertensiana* | EU391333 | EF525704 |
| 11 | *Corallorhiza odontorhiza* | EU391326 | EF525701 |
| 12 | *Corallorhiza striata var. striata* | EU391349 | EF525702 |
| 13 | *Corallorhiza striata var. vreelandii* | EU391352 | EF525705 |
| 14 | *Corallorhiza trifida* | EU391324 | EF525695 |
| 15 | *Corallorhiza wisteriana* | EU391327 | EF525703 |
| 16 | *Cremastra aphylla* | LC176594 | LC176614 |
| 17 | *Cremastra appendiculata* | KM526764 | JX293182 |
| 18 | *Cremastra unguiculata* | LC176593 | LC176616 |
| 19 | *Cremastra variabilis* | LC176595 | LC176615 |
| 20 | *Dactylostalix ringens* | LC176596 | LC176617 |
| 21 | *Danxiaorchis mangdangshanensis* | OP104024 | OP122564 |
| 22 | *Danxiaorchis singchiana* | - | MN990438 |
| 23 | *Danxiaorchis yangii 1* | KY968693 | KY968689 |
| 24 | *Danxiaorchis yangii 2* | KY968694 | KY968690 |
| 25 | *Danxiaorchis yangii 3* | KY968695 | KY968691 |
| 26 | *Danxiaorchis yangii 4* | KY968696 | KY968692 |
| 27 | *Ephippianthus sawadanus* | LC176598 | LC176619 |
| 28 | *Ephippianthus schmidtii* | LC176599 | LC176620 |
| 29 | *Govenia liliacea* | AF521056 | AY121723 |
| 30 | *Govenia sp.* | EF525672 | EF525690 |
| 31 | *Govenia utriculata* | LC176600 | LC176621 |
| 32 | *Tipularia cunninghamii* | LC176597 | LC176618 |
| 33 | *Tipularia discolor* | LC176606 | LC176628 |
| 34 | *Tipularia japonica* | LC176607 | LC176629 |
| 35 | *Tipularia szechuanica* | LC176605 | LC176627 |
| 36 | *Yoania amagiensis* | LC176610 | - |
| 37 | *Yoania flava* | LC176608 | - |
| 38 | *Yoania japonica* | LC176609 | - |
| 39 | *Yunorchis pingbianensis* | KM526768 | KM526763 |

Table S2. GenBank information for the taxa used in the present study (plastid genome)

|  | **Tribe** | **Sub-tribe** | **Species** | **GenBank accession number** |
| --- | --- | --- | --- | --- |
| 1 | *Epidendreae* | *Calypsoinae* | *Calypso bulbosa var. occidentalis* | MG874037 |
| 2 |  |  | *Corallorhiza bentleyi* | MG874035 |
| 3 |  |  | *Corallorhiza bulbosa* | KM390013 |
| 4 |  |  | *Corallorhiza macrantha* | KM390017 |
| 5 |  |  | *Corallorhiza mertensiana* | KM390018 |
| 6 |  |  | *Corallorhiza odontorhiza* | KM390021 |
| 7 |  |  | *Corallorhiza striata* | MG874034 |
| 8 |  |  | *Corallorhiza trifida* | MG874036 |
| 9 |  |  | *Corallorhiza wisteriana* | KM390020 |
| 10 |  |  | *Cremastra appendiculata* | MG925366 |
| 11 |  |  | *Danxiaorchis singchiana*1 | MN584923 |
| 12 |  |  | *Danxiaorchis singchiana 2* | MN990438 |
| 13 |  |  | *Danxiaorchis mangdangshanensis* | OP122564 |
| 14 |  | *Laeliinae* | *Cattleya crispata* | KP168671 |
| 15 |  |  | *Cattleya liliputana* | KP202881 |
| 16 |  | *Pleurothallidinae* | *Anathallis obovata* | MH979332 |
| 17 |  |  | *Masdevallia coccinea* | KP205432 |
| 18 |  |  | *Masdevallia picturata* | KJ566305 |
| 19 | *Vandeae* | *Aeridinae* | *Neofinetia falcata* | KT726909 |
| 20 | *Collabieae* |  | *Calanthe triplicata* | KF753635 |
